# Integrating the Urea-to-Creatinine Ratio with Clinical Variables to Improve Etiological Discrimination in Acute Kidney Injury

**DOI:** 10.3390/jcm15176604

**Published:** 2026-08-26

**Authors:** Luca Malatesta, Marco Allinovi, Sofia Brucia, Mariapia Carafa, Sara Dal Lago, Glenda Cancila, Alberto Parise, Chiara Giannini, Francesco Peyronel

**Affiliations:** 1 Department of Experimental and Clinical Biomedical Sciences “Mario Serio”, University of Florence, 50134 Florence, Italy; lucamalatesta1993@gmail.com (L.M.); sofia.brucia@unifi.it (S.B.); mariapia.carafa@unifi.it (M.C.); sara.dallago@unifi.it (S.D.L.); chiara.giannini@unifi.it (C.G.); 2 Nephrology, Dialysis and Transplant Unit, Careggi University Hospital, 50134 Florence, Italy; 3 Nephrology and Dialysis Unit, San Giuseppe Hospital, 50053 Empoli, Italy; glenda.cancila@uslcentro.toscana.it; 4 Department of Internal Medicine, Parma University Hospital, 43126 Parma, Italy; aparise@ao.pr.it; 5 Department of Experimental and Clinical Medicine, University of Florence, 50134 Florence, Italy; francesco.peyronel@unifi.it; 6 Clinical Research Center for Rare Diseases Aldo e Cele Daccò, Istituto di Ricerche Farmacologiche Mario Negri IRCCS, 24020 Ranica, Bergamo, Italy

**Keywords:** acute kidney injury, urea-to-creatinine ratio, AKI, BUN, pre-renal AKI, parenchymal AKI, post-renal AKI

## Abstract

**Background**: The urea-to-creatinine ratio (UCR) is used to support the differential diagnosis of acute kidney injury (AKI); however, its diagnostic utility remains debated. We explored the clinical relevance of UCR in the initial etiological assessment of AKI, also considering relevant clinical modifiers. **Methods**: We retrospectively analyzed 590 hospitalized patients classified as pre-renal (n = 319), parenchymal (n = 135), or post-renal (n = 136) AKI. UCR was compared across AKI subtypes and stratified by pre-existing chronic kidney disease (CKD). Logistic regression models assessed the association between UCR and pre-renal AKI, excluding post-renal cases. Diagnostic performance was evaluated using receiver operating characteristic curve analysis. An exploratory analysis was performed within parenchymal AKI according to histopathological patterns and the presence of nephrotic syndrome. **Results**: Baseline characteristics differed across AKI subtypes, including age, sex, and CKD. UCR was higher in pre-renal than in parenchymal and post-renal AKI (*p* < 0.001), despite substantial overlap. Stratification by CKD did not reveal differences within AKI subtypes; however, in pre-renal AKI, UCR decreased progressively with advancing CKD stage (*p* < 0.001). UCR was significantly associated with pre-renal AKI, and diagnostic performance improved when combined with age and advanced CKD. Within parenchymal AKI, higher UCR values were observed in glomerular disease, particularly with nephrotic syndrome. **Conclusions**: UCR varies across AKI etiologies and is influenced by age and baseline kidney function. Although its standalone diagnostic performance is limited, integration with simple clinical variables improves discrimination between pre-renal and parenchymal AKI, supporting its use as a complementary tool in early AKI evaluation while emphasizing the need for comprehensive clinical assessment.

## 1. Introduction

Acute kidney injury (AKI) is a common and multifaceted clinical condition characterized by an abrupt decline in renal function occurring over hours to days. It is classically classified into pre-renal, intrinsic or parenchymal, and post-renal forms, reflecting distinct underlying pathophysiological mechanisms such as reduced effective circulating volume, structural kidney injury, or urinary tract obstruction, respectively. AKI is associated with substantial morbidity and mortality and remains a frequent cause of hospitalization across medical specialties [1]. In emergency medicine and internal medicine settings, early etiological assessment of AKI is critical to guide timely management decisions, yet often relies on limited laboratory information available at presentation.

According to KDIGO guidelines, AKI is diagnosed and staged based on changes in serum creatinine and urine output [2,3]. Although blood urea levels are not included in the formal diagnostic criteria, they have long been used in clinical practice to assess renal dysfunction and metabolic disturbances. Urea and creatinine are both excreted by the kidney but originate from different metabolic pathways and exhibit distinct renal handling. Creatinine is produced at a relatively constant rate from muscle metabolism and primarily reflects glomerular filtration, whereas urea derives from hepatic amino acid catabolism and undergoes variable tubular reabsorption influenced by neurohormonal activation, effective circulating volume, and protein metabolism.

Since the mid-20th century, the balance between urea and creatinine production and excretion has been recognized as a potentially informative parameter in renal disease [4]. The circulating level of urea, the end product of hepatic amino acid catabolism, depends on both production and renal handling, including variable tubular reabsorption influenced mainly by volume status. In contrast, creatinine, which is generated from muscle metabolism at a relatively constant rate, is primarily eliminated by glomerular filtration. As a result, in states of renal hypoperfusion, urea rises disproportionately relative to creatinine, forming the physiological basis of the urea-to-creatinine ratio (UCR) in blood [4]. Traditionally, an elevated UCR, or blood urea nitrogen-to-creatinine ratio (BCR), has been interpreted as suggestive of pre-renal AKI, reflecting enhanced tubular urea reabsorption in states of hypovolemia or reduced renal perfusion. In these settings, activation of the renin–angiotensin–aldosterone system, sympathetic nervous system, and arginine vasopressin promotes preferential urea retention, leading to a disproportionate rise in urea relative to creatinine [5].

Beyond renal hypoperfusion, elevated UCR has also been described in upper gastrointestinal bleeding, where digestion and absorption of blood proteins increase systemic urea generation independently of kidney function. Accordingly, UCR has been extensively studied in gastrointestinal emergencies as a potential marker of bleeding source and disease severity [6,7,8,9,10,11,12]. Similarly, a substantial body of literature has investigated UCR or BCR in heart failure and cardiogenic shock, primarily focusing on prognostic outcomes such as mortality and rehospitalization, rather than etiological implications [13,14,15,16,17,18,19]. Several other non-renal factors may limit UCR diagnostic specificity. An increase in urea production affecting UCR may develop in conditions such as corticosteroid therapy, high dietary protein intake, prolonged fasting or treatment with drugs such as tetracyclines, whereas liver disease may reduce urea synthesis. In addition, UCR may be affected by changes in creatinine generation, including rhabdomyolysis or creatine supplementation, potentially leading to misleading values independent of renal function. In renal cohorts, data remain scarce and often contradictory [20,21,22,23,24,25,26].

Many studies exclude patients with chronic kidney disease (CKD), fail to stratify by baseline renal function, or focus exclusively on selected clinical scenarios. Moreover, the diagnostic value of UCR across the full spectrum of AKI etiologies (i.e., pre-renal, parenchymal, and post-renal) has rarely been systematically explored in large, unselected populations. Importantly, no prior studies have comprehensively examined UCR behavior within parenchymal AKI according to histopathological patterns, such as glomerular vs. tubulo-interstitial injury, or clinical phenotypes including nephrotic syndrome. Similarly, the modifying effects of age and CKD stage on UCR interpretation in AKI remain poorly defined. As a result, the practical role of UCR in contemporary acute care is uncertain, and its integration into modern diagnostic pathways has been largely abandoned.

The aim of this study was to explore the clinical significance of UCR as a supportive laboratory parameter in the initial etiological assessment of AKI in hospitalized patients, while evaluating potential clinical modifiers, including age, pre-existing CKD, and gastrointestinal bleeding. By analyzing a large hospital-based cohort with detailed clinical and histopathological characterization, we sought to clarify the contextual value of UCR across AKI subtypes and to assess its performance when integrated with simple clinical variables.

## 2. Materials and Methods

### 2.1. Study Design and Population

We conducted a retrospective observational study at Careggi University Hospital (Florence, Italy), reviewing electronic medical records from the Emergency-Urgency Department, Internal Medicine, and Nephrology wards, together with the institutional renal biopsy registry. Potentially eligible patients were identified using a structured keyword search within admission and discharge diagnoses (the complete list of search terms is reported in Supplementary Table S1). Individual medical records were subsequently reviewed to confirm eligibility and to ascertain AKI etiology.

Patients were included if they:

(1) Fulfilled KDIGO criteria for AKI [2];
(2) Had available measurements of both serum urea and creatinine, obtained at the time of AKI recognition (corresponding to hospital admission for community-acquired cases or to the first biochemical evidence of AKI during hospitalization);
(3) Had sufficient clinical documentation to allow etiological classification of AKI.

Patients were excluded if they:

(1) Did not fulfill KDIGO AKI criteria;
(2) Lacked admission urea or creatinine measurements;
(3) Had insufficient clinical data for etiological assessment of AKI;
(4) Presented with overlapping or mixed causes of AKI (e.g., combined pre-renal and parenchymal AKI, sepsis-related AKI);
(5) Had intrinsic AKI without histological confirmation by kidney biopsy.

### 2.2. AKI Classification

AKI was classified into mutually exclusive etiological categories based on integrated clinical, laboratory, imaging, and histopathological data:

- Pre-renal AKI, defined by a clinical context consistent with reduced renal perfusion due to hypovolemia or reduced effective circulating volume (e.g., gastrointestinal fluid losses, bleeding, poor oral intake, or heart failure), in the absence of evidence supporting parenchymal kidney disease or urinary tract obstruction;
- Parenchymal (intrinsic) AKI, confirmed by laboratory tests and renal biopsy;
- Post-renal AKI, documented by imaging evidence of urinary tract obstruction.

Cases were retrospectively classified as pre-renal, parenchymal, or post-renal AKI by the nephrologists participating in the study, based on review of the complete electronic medical records. Etiological classification was based on an integrated assessment of all clinical information available at the time of AKI diagnosis, including recent medical history, physical examination findings, kidney and urinary tract imaging, and, when applicable, histopathological findings from kidney biopsy. Particular attention was paid to identifying the predominant mechanism responsible for AKI, and only patients with a single, clearly identifiable etiology were included, whereas patients with mixed or overlapping causes of AKI were excluded. During the adjudication process, the calculated UCR value, which represented the study variable, was not available to the reviewing nephrologists and therefore did not contribute to etiological classification. Etiological classification was based exclusively on the routine clinical assessment performed using the available medical history, physical examination, laboratory findings, imaging studies, and, when applicable, kidney biopsy results. Each case was evaluated by a single nephrologist according to the predefined study criteria. As each patient underwent a single adjudication, formal duplicate adjudication and assessment of inter-rater reliability were not performed.

### 2.3. Laboratory Measurements and Clinical Parameters

Serum urea and creatinine values (mg/dL) used for UCR calculation were obtained from the same blood sample collected at the time of AKI recognition. For community-acquired AKI, this corresponded to the first laboratory assessment performed at hospital admission, whereas for hospital-acquired AKI, measurements corresponded to the first blood sample showing biochemical evidence of AKI according to KDIGO criteria. UCR (mg/mg) was calculated as urea divided by creatinine. Age, sex, previous CKD status, and CKD stage were also collected. Pre-existing CKD was defined according to documented baseline eGFR or prior records and staged following KDIGO criteria [27]. Advanced CKD was defined as stage 4–5. Within the parenchymal AKI group, histopathological diagnoses were categorized as glomerular or tubulo-interstitial injury. Glomerular diseases were further stratified according to the presence of nephrotic syndrome (i.e., proteinuria > 3.5 g/24 h and serum albumin ≤ 2.5 g/dL).

UCR is frequently expressed with the molar ratio (mol/mol) or, alternatively, as a mass ratio of urea to creatinine (mg/mg). However, the Anglo-Saxon literature frequently reports the BCR, expressed as mg/mg, which reflects only the nitrogen component of urea rather than total urea concentration.

In our study we expressed the UCR as mg/mg, but to help translate the results into clinical practice, we report the conversion formulas below.

Conversion from mass-based UCR to molar UCR: UCR (mol/mol) = UCR (mg/mg) × 1.88

Conversion from mass-based UCR to BCR: BCR (mg/mg) = UCR (mg/mg) × 0.47

Conversion from BCR to molar UCR: UCR (mol/mol) = BCR (mg/mg) × 4.03

### 2.4. Statistical Analysis

Continuous variables are reported as median and interquartile range (IQR), and categorical variables as count or proportion and percentage. Differences between groups were assessed using the Mann–Whitney U test or the Kruskal–Wallis test for continuous variables and Fisher’s exact test for categorical variables. When overall significance was detected, pairwise comparisons were performed using Dunn’s multiple comparisons test.

Logistic regression models were developed to assess the association between UCR and the diagnosis of pre-renal AKI, excluding post-renal cases. Post-renal AKI was excluded from regression modeling and diagnostic performance analyses, as the diagnosis of urinary tract obstruction relies primarily on imaging and not on biochemical markers. Sequential models included:

(1) Univariate UCR;
(2) Age-adjusted UCR;
(3) Multivariate models incorporating age and advanced CKD (stage 4–5).

Odds ratios (ORs) are reported with their respective 95% confidence intervals (95%CIs).

Model discrimination was evaluated using receiver operating characteristic (ROC) curves and area under the curve (AUC). Optimal probability thresholds were identified using the Youden index, with corresponding sensitivity, specificity, positive predictive value, negative predictive value, and accuracy. Model performance was compared using the likelihood ratio test (LRT) for nested models.

An exploratory subgroup analysis was performed within parenchymal AKI to compare UCR across histopathological patterns and clinical phenotypes.

All analyses and plots were performed using R software (version 4.5.1; R Foundation for Statistical Computing, Vienna, Austria) within the RStudio environment (version 2026.01.2; Posit Software, Boston, MA, USA) and GraphPad Prism software (version 9; GraphPad Software, San Diego, CA, USA). A two-sided *p*-value < 0.05 was considered statistically significant.

## 3. Results

### 3.1. Study Population and Baseline Characteristics

A total of 590 patients with AKI were included in the study cohort. Baseline characteristics are summarized in Table 1 and Supplementary Table S2. Median age at AKI recognition was 77 years (IQR 64–85 years), and 64.1% of patients were male. According to clinical classification, 319 patients (54.1%) had pre-renal AKI, 135 (22.9%) parenchymal AKI, and 136 (23.1%) post-renal AKI. Baseline characteristics differed significantly across AKI subtypes. Patients with pre-renal and post-renal AKI were older, whereas parenchymal AKI occurred at a younger age (*p* < 0.001). Male sex was markedly prevalent in post-renal AKI (*p* < 0.001). Pre-existing CKD (i.e., stage ≥ 3) was present in 46.5% of cases and was more frequent in pre-renal and post-renal AKI than in parenchymal AKI (*p* = 0.006). Advanced CKD (stages 4–5) was uncommon in patients with parenchymal AKI, with no patients with CKD stage 5 observed in this group.

### 3.2. UCR According to AKI Etiology

**Figure 1:**
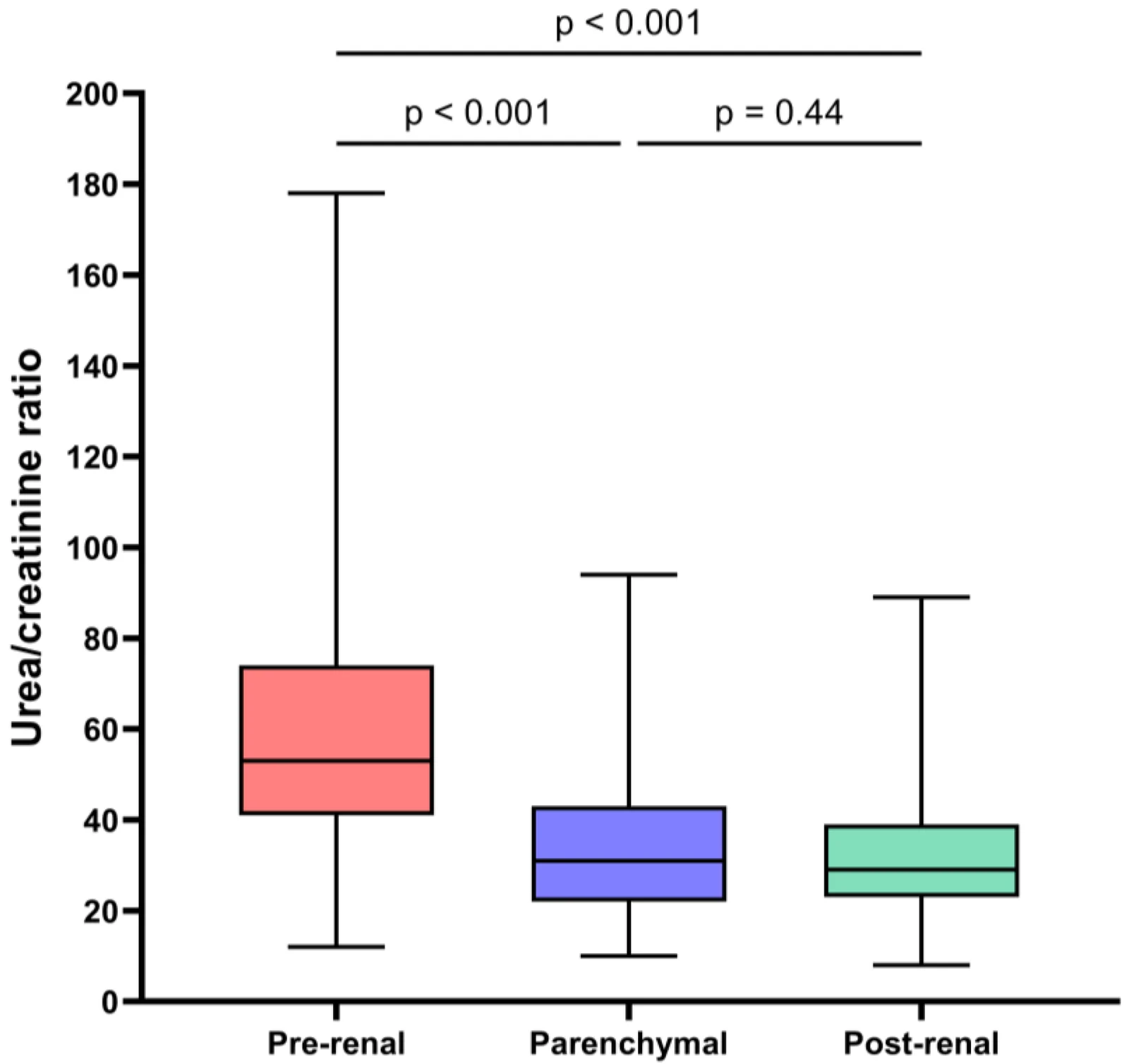
Distribution of urea-to-creatinine ratio (UCR) values across AKI etiologies.

UCR differed significantly according to AKI etiology (*p* < 0.001—Figure 1, Table 2). Patients with pre-renal AKI showed higher UCR values compared with those with parenchymal and post-renal AKI (median [IQR]: 53.5 [41–73.5] vs. 31 [23–42.5] vs. 29 [23.5–38.5], respectively). No significant difference was observed between parenchymal and post-renal AKI. Despite these differences, substantial overlap in UCR values was observed across groups, with approximately one-quarter of patients with pre-renal AKI displaying UCR values within the range observed in parenchymal AKI.

### 3.3. Impact of CKD and Gastrointestinal Bleeding on UCR

Stratification by pre-existing CKD as a binary variable did not reveal significant differences in UCR within any AKI subtype (Table 2). However, among patients with pre-renal AKI, UCR values decreased progressively with advancing CKD (*p* < 0.001), indicating a graded effect of baseline renal function on UCR behavior (Table 2). Among patients with pre-renal AKI, those with concomitant gastrointestinal bleeding exhibited higher UCR (*p* < 0.001). However, substantial overlap in UCR values was observed between groups (Table 2).

### 3.4. Association and Discrimination of UCR for Pre-Renal AKI

**Figure 2:**
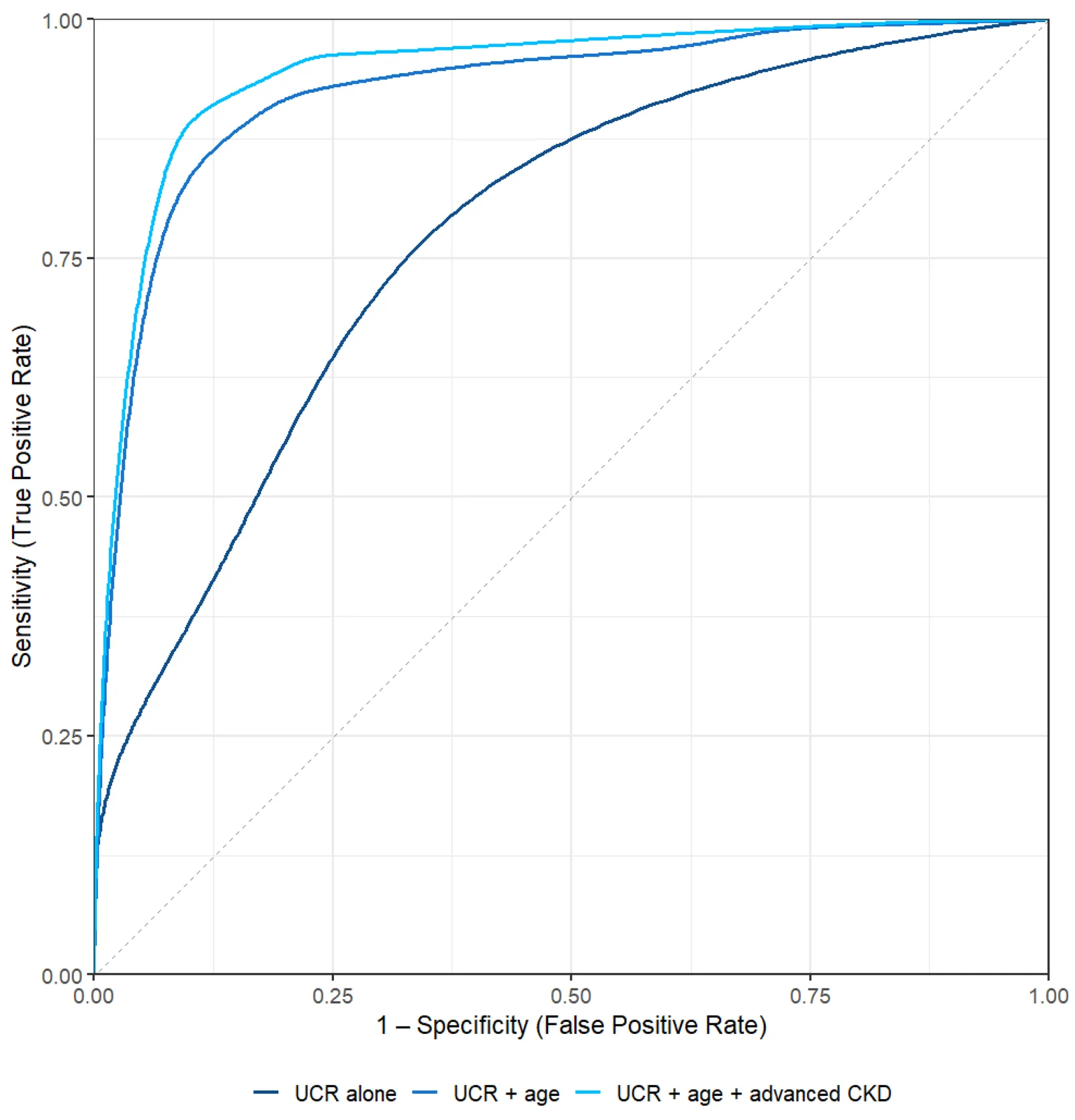
ROC analysis comparing UCR-based models (UCR alone and in combination with clinical variables) for the identification of pre-renal AKI. The dotted diagonal line represents the line of no discrimination (AUC = 0.5).

In univariate logistic regression comparing pre-renal and parenchymal AKI, UCR was significantly associated with pre-renal AKI (OR per unit increase 1.069, 95%CI 1.053–1.086; *p* < 0.001—Table 3). ROC curve analysis yielded an AUC of 0.794, indicating modest discriminative performance (Figure 2), consistent with the substantial overlap observed between AKI subtypes. Age was also strongly associated with pre-renal AKI, whereas the association with CKD of any stage was attenuated after age adjustment. In contrast, advanced CKD (stages 4–5) retained a strong independent association with pre-renal AKI (Table 3). An age-adjusted model combining UCR and age showed higher in-sample discrimination (AUC 0.939). Further inclusion of advanced CKD was associated with higher discrimination (AUC 0.959) (Figure 2). Additional diagnostic performance metrics, including Youden-derived thresholds, sensitivity, specificity, and predictive values, are provided in Supplementary Table S3. Model comparison using LRT confirmed a significantly improved fit of the extended model including advanced CKD compared with the age-adjusted model (*p* < 0.001).

### 3.5. Exploratory Analysis in Parenchymal AKI

**Figure 3:**
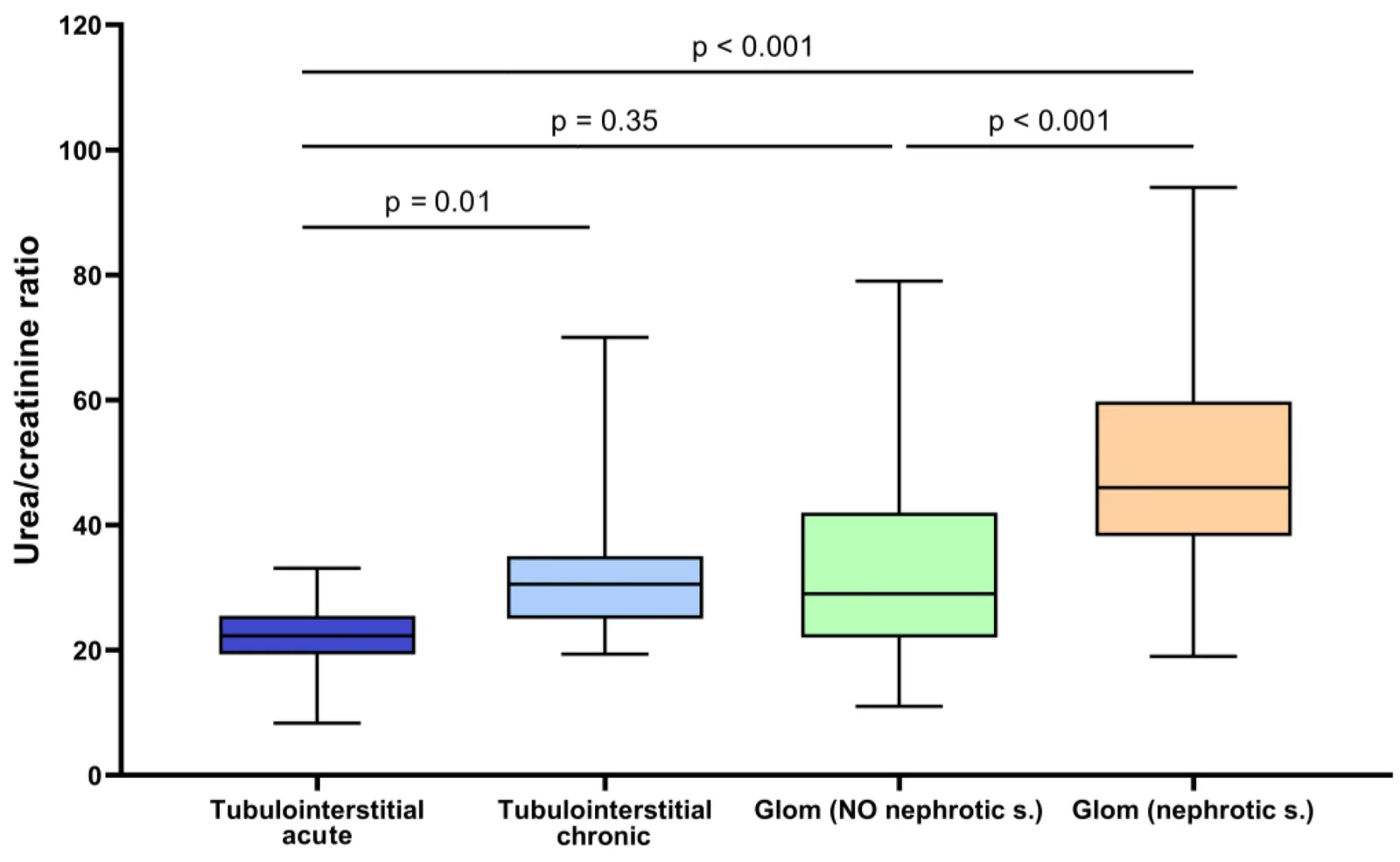
Distribution of urea-to-creatinine ratio (UCR) values across histopathological subgroups of intrinsic AKI (tubulo-interstitial nephritis, nephrotic glomerular disease, non-nephrotic glomerular disease).

Within parenchymal AKI, UCR values differed significantly according to underlying histopathological patterns. Patients with glomerular disease exhibited higher UCR values than those with tubulo-interstitial injury (median [IQR]: 36 [26–47] vs. 26.5 [21–31]; *p* < 0.001—Table 2). Among glomerular AKI, the presence of nephrotic syndrome was associated with higher UCR values compared with non-nephrotic glomerular disease (median [IQR]: 46 [38.5–60] vs. 29 [22–42]; *p* < 0.001). UCR values in non-nephrotic glomerular disease largely overlapped with those observed in tubulo-interstitial AKI (Table 2, Figure 3). However, among patients with biopsy-proven tubulo-interstitial AKI, those with acute tubulo-interstitial nephritis showed significantly lower UCR values compared with those with chronic tubulo-interstitial nephritis (median [IQR]: 23 [20–26] vs. 30 [25.5–35]; *p* = 0.01).

## 4. Discussion

UCR, or BCR (which can be obtained by simply dividing UCR by 2.14), has long been considered an adjunctive marker in the evaluation of pre-renal AKI. However, evidence in renal cohorts remains limited and often contradictory [20,21,22,23,24,25,26], and its diagnostic specificity has been increasingly questioned. In recent years, UCR has been more extensively investigated in non-renal settings, particularly heart failure and gastrointestinal bleeding, where it has emerged primarily as a prognostic marker, reflecting systemic stress and hemodynamic compromise rather than a specific indicator of renal injury [6,7,8,9,10,11,12,13,14,15,16,17,18,19].

In this large hospital-based cohort, UCR values differed significantly across AKI etiologies, with higher levels observed in pre-renal AKI compared with parenchymal and post-renal forms. These findings are consistent with the established physiological paradigm of enhanced tubular urea reabsorption during renal hypoperfusion [5] and reduced tubular urea reabsorption in obstructive conditions (both at the tubular and urinary tract level) [28]. Nevertheless, substantial overlap between groups was observed, underscoring the limited utility of UCR as a standalone discriminator and confirming prior reports questioning its diagnostic accuracy when used in isolation. Accordingly, an elevated UCR may support the etiological assessment of pre-renal AKI, whereas a normal UCR does not exclude this diagnosis.

A central finding of our study is that the diagnostic performance of UCR improves markedly when interpreted within its clinical context. While UCR alone showed only modest discrimination, integration with age and advanced CKD substantially increased discrimination within our cohort. This highlights that UCR does not function as an independent diagnostic marker, but rather as a context-sensitive parameter, whose interpretation must account for patient-related modifiers. Older age and reduced renal reserve appear to amplify vulnerability to hemodynamic stress, thereby enhancing the discriminatory value of UCR in identifying pre-renal AKI. Baseline kidney function emerged as a key modifier of UCR behavior. Although stratification by CKD as a binary variable did not affect UCR within AKI subtypes, a graded reduction in UCR was observed with advancing CKD stage among patients with pre-renal AKI. This suggests that preserved renal function enables a more effective adaptive tubular response to renal hypoperfusion, whereas CKD attenuates these mechanisms, potentially leading to underestimation of pre-renal physiology when relying on UCR alone. In addition, several determinants of urea and creatinine metabolism, including liver disease, nutritional status, corticosteroid therapy, and heart failure, were not systematically available and therefore could not be accounted for. Residual confounding from these clinical factors and other unmeasured variables therefore cannot be excluded. Among other things, current KDIGO CKD guidelines recommend a low-protein diet (approximately 0.6–0.8 g/kg/day) for many patients with advanced CKD not receiving kidney replacement therapy to preserve kidney function by slowing eGFR decline. Reduced protein intake may decrease hepatic urea production, resulting in lower circulating urea concentrations independently of tubular urea handling. Consequently, the lower UCR observed in patients with CKD stage 4–5 may reflect both impaired tubular urea reabsorption and reduced urea production related to dietary protein restriction, although the relative contribution of these mechanisms could not be determined in the present study. These findings emphasize the need for caution when interpreting UCR in patients with severely reduced renal reserve.

As expected, concomitant gastrointestinal bleeding was associated with higher UCR values in pre-renal AKI, reflecting increased urea generation and absorption. However, marked overlap between patients with and without bleeding limits the clinical specificity of this association, reinforcing that UCR elevations in this setting should be interpreted alongside the broader clinical picture. Post-renal AKI was characterized by relatively low UCR values, comparable to those observed in parenchymal AKI, confirming the limited diagnostic utility of UCR in obstructive uropathy and underscoring the central role of imaging in this context.

An exploratory analysis within parenchymal AKI revealed distinct UCR patterns according to histopathological injury. Glomerular disease was associated with higher UCR values than tubulo-interstitial injury, particularly in the presence of nephrotic syndrome. Conversely, non-nephrotic glomerular disease showed UCR values largely overlapping with tubulo-interstitial AKI. These observations extend prior reports and suggest that UCR may reflect heterogeneous pathophysiological mechanisms within intrinsic AKI, potentially influenced by hormonal and hemodynamic adaptations in nephrotic states. A recent prospective study proposed a low BCR as a diagnostic marker for biopsy-proven acute interstitial nephritis [26]; however, this finding was derived from a small cohort and showed only moderate discrimination, underscoring that BCR alone is unlikely to provide sufficient diagnostic accuracy in intrinsic AKI. Overall, our data show substantial overlap across parenchymal phenotypes, emphasizing the need to integrate biochemical indices with clinical and histopathological assessment.

Several limitations warrant consideration. The retrospective, single-center design limits the generalizability of our findings. The multivariable analyses were exploratory and were intended to examine the contextual contribution of readily available clinical variables to UCR interpretation, rather than to derive a diagnostic prediction score or clinical decision tool. Accordingly, the reported AUCs represent apparent discrimination within the study cohort and were not corrected for optimism or internally validated; their magnitude should therefore not be interpreted as an estimate of performance in independent patients or as evidence of clinical benefit. Prospective studies specifically designed for diagnostic model development and validation would be required before these findings could be translated into a clinical decision tool. Furthermore, the exclusion of patients with mixed AKI etiologies and the restriction of parenchymal AKI to biopsy-proven cases may have resulted in a selected study population with relatively well-defined etiological phenotypes. These design features may have increased the separation between pre-renal and parenchymal AKI and, consequently, contributed to the high apparent discrimination observed in our cohort. Therefore, the reported AUCs may not be directly applicable to unselected populations, in which mixed etiologies are common. An additional limitation relates to the retrospective etiological classification of AKI. Pre-renal AKI lacks a universally accepted objective reference standard, and diagnosis necessarily relied on integrated clinical judgment based on medical history, physical examination, laboratory findings, and imaging. Although the calculated UCR value was not available during adjudication and therefore could not directly influence etiological classification, some degree of misclassification cannot be excluded, particularly in borderline cases. Parenchymal AKI was restricted to biopsy-proven cases, introducing potential selection bias toward more severe or atypical presentations. While this strategy increases diagnostic accuracy and reduces the risk of outcome misclassification, it also results in a highly selected population that may not be representative of patients with intrinsic AKI in clinical practice, where histological confirmation is rare, and diagnosis is usually based on clinical, laboratory tests, and imaging findings.

Despite these limitations, the large sample size, detailed clinical phenotyping, stratification by CKD stage, and integration of histopathological data represent key strengths.

## 5. Conclusions

In conclusion, UCR exhibits distinct patterns across AKI etiologies but demonstrates substantial overlap that limits its standalone diagnostic value. Its discriminatory value within our cohort increased when interpreted alongside simple clinical variables, particularly age and baseline kidney function. These findings support a context-dependent interpretation of UCR as a complementary parameter in the early etiological assessment of AKI, rather than its use as an isolated diagnostic marker. Comprehensive clinical evaluation remains essential for determining the underlying cause of AKI.

## Figures and Tables

**Table 1:** Main characteristics of the study cohort and comparison depending on AKI type.

Variable	Overall (N = 590)	Pre-Renal AKI (n = 319)	Parenchymal AKI (n = 135)	Post-Renal AKI (n = 136)	*p*-Value
Age, *years*	77 (64–85)	82 (75–87)	56 (48–67)	76.5 (67–87)	<0.001
Female sex	212 (35.9)	143 (44.8)	48 (35.6)	21 (15.4)	<0.001
Creatinine, *mg/dL*	2.7 (1.9–4.5)	2.4 (1.8–3.5)	2.5 (1.8–4.3)	5.2 (2.3–8.4)	<0.001
Urea, *mg/dL*	130 (89–192)	140 (100–207.5)	93 (67.5–136)	140 (87–230)	<0.001
Previous CKD	263/565 (46.5)	154/310 (49.7)	41/121 (33.9)	68/134 (50.7)	0.006
Stage 3 (any)	163/263 (62.0)	92/154 (59.7)	33/37 (89.2)	38/66 (57.6)	
Stage 3A	53/132 (40.2)	27/74 (36.5)	21/33 (63.6)	15/38 (39.5)	
Stage 3B	79/132 (59.8)	47/74 (63.5)	12/33 (36.4)	23/38 (60.5)	
Stage 4	71/244 (29.1)	53/154 (34.4)	4/37 (10.8)	14/66 (21.2)	
Stage 5	23/244 (9.4)	9/154 (5.8)	0/37 (0.0)	14/66 (21.2)	

Continuous variables are presented as median (IQR), and categorical variables as count (%) or proportion (%). Differences between groups were assessed using the Kruskal–Wallis test for continuous variables and Fisher’s exact test for categorical variables. When overall significance was detected, pairwise comparisons were performed using Dunn’s multiple comparisons test (see Supplementary Table S1).

**Table 2:** Urea/creatinine ratio in AKI: comparison across etiologies and clinical modifiers.

Comparison	Subgroup	n	Median (IQR)	*p*-Value
AKI overall	Pre-renal	319	53.5 (41–73.5)	<0.001
	Parenchymal	135	31 (23–42.5)	
	Post-renal	136	29 (23.5–38.5)	
	Pre-renal vs. parenchymal			<0.001
	Pre-renal vs. post-renal			<0.001
	Parenchymal vs. post-renal			0.440
AKI stratified by previous CKD	Pre-renal CKD+	154	52 (41–70)	0.196
	Pre-renal CKD−	156	54 (40.5–79)	
	Parenchymal CKD+	41	28 (22–44)	0.581
	Parenchymal CKD−	80	31 (25–43)	
	Post-renal CKD+	68	27.5 (21–38)	0.147
	Post-renal CKD−	66	29.5 (25–39)	
Pre-renal AKI stratified by previous CKD stage	CKD stage 3	92	55.5 (45–75)	<0.001
	CKD stage 4	53	48 (35.5–63)	
	CKD stage 5	9	37 (29–39.5)	
	CKD stage 3 vs. stage 4			0.020
	CKD stage 3 vs. stage 5			0.002
	CKD stage 4 vs. stage 5			0.120
Parenchymal AKI stratified by previous CKD stage	CKD stage 3	33	29 (22–44.5)	0.753
	CKD stage 4	4	28 (21.5–41.5)	
	CKD stage 5	-	-	
Post-renal AKI stratified by previous CKD stage	CKD stage 3	38	27 (22–35.5)	0.330
	CKD stage 4	14	38 (21–44.5)	
	CKD stage 5	14	23.5 (17–33.5)	
Pre-renal AKI stratified by gastrointestinal bleeding	Gastrointestinal bleeding +	91	64 (48–82)	
	Gastrointestinal bleeding −	228	51 (39–68)	
Parenchymal AKI stratified by tissue injury at kidney biopsy	Glomerular	91	36 (26–47)	<0.001
	Tubulo-interstitial	44	26.5 (21–31)	
	Glomerular with nephrotic syndrome	32	46 (38.5–60)	<0.001
	Glomerular without nephrotic syndr.	59	29 (22–42)	
	Tubulo-interstitial	44	26.5 (21–31)	

Differences between groups were assessed using the Kruskal–Wallis test, followed by Dunn’s multiple comparisons test. Comparisons between two subgroups (e.g., presence vs. absence of previous CKD, or gastrointestinal bleeding) were performed using the Mann–Whitney U test.

**Table 3:** Logistic regression models evaluating the association between UCR and pre-renal AKI.

Variable	Univariate Models		Age-Adjusted Models		Multivariate Model	
	OR (95%CI)	*p*-Value	OR (95%CI)	*p*-Value	OR (95%CI)	*p*-Value
UCR (per unit)	1.069 (1.053–1.086)	<0.001	1.053 (1.036–1.074)	<0.001	1.067 (1.031–1.113)	<0.001
Age (per year)	1.144 (1.117–1.175)	<0.001	-	-	1.168 (1.109–1.247)	<0.001
Female sex (vs. male)	1.473 (0.975–2.242)	0.068				
Pre-existing CKD (stage ≥3)	1.926 (1.250–3.002)	0.003	1.349 (0.741–2.474)	0.328		
Pre-existing advanced CKD (stage 4–5)	5.56 (2.082–19.334)	0.002	24.388 (4.419–228.96)	0.001	32.285 (5.651–303.843)	<0.001

## Data Availability

The data underlying this article will be shared upon reasonable request made to the corresponding author.

## Supplementary Materials

The following supporting information can be downloaded at: https://www.mdpi.com/article/10.3390/jcm15176604/s1, Table S1. Complete list of search terms used for patient identification; Table S2. Main characteristics of the study cohort and comparison depending on AKI type; Table S3. Model comparison.
