# Supplementary material for: Integrating the Urea-to-Creatinine Ratio with Clinical Variables to Improve Etiological Discrimination in Acute Kidney Injury
Source: J Clin Med. 2026 Aug 26;15(17):6604. doi: 10.3390/jcm15176604 (PMC13566869; doi:10.3390/jcm15176604)
Supplement: Supplementary file 1 [file jcm-15-06604-s001.zip › jcm-4435129-supplementary.pdf]

Supplementary Table S1. Complete list of search terms used for patient identification

AKI<br>Acute kidney injury<br>Acute renal failure<br>Renal failure<br>Pre-renal<br>Post-renal<br>Hypovolemia<br>Dehydration	Vomiting<br>Diarrhea<br>Heart failure<br>Obstruction<br>Hydronephrosis<br>Bladder distension<br>Kidney stone<br>Neobladder	Glomerulonephritis<br>Nephrotic syndrome<br>Nephritic syndrome<br>Nephritis<br>Tubular necrosis<br>Vasculitis<br>Lupus<br>HUS	Myeloma<br>Cast<br>Amyloidosis<br>Bleeding<br>Melena<br>Angiodysplasia<br>Hematemesis<br>Ulcer

Supplementary Table S2. Main characteristics of the study cohort and comparison depending on AKI type

Variable	Overall (N=590)	Pre-renal AKI (n=319)	Parenchymal AKI (n=135)	Post-renal AKI (n=136)	p-value
Age, *years*	77 (64-85)<br><br>Pre-renal vs parenchymal<br>Pre-renal vs post-renal<br>Parenchymal vs post-renal	82 (75-87)	56 (48-67)	76.5 (67-87)	<0.001<br><br><0.001<br>0.002<br><0.001
Female sex	212 (35.9)<br><br>Pre-renal vs parenchymal<br>Pre-renal vs post-renal<br>Parenchymal vs post-renal	143 (44.8)	48 (35.6)	21 (15.4)	<0.001<br><br>0.077<br><0.001<br><0.001
Creatinine, *mg/dL*	2.7 (1.9-4.5)<br><br>Pre-renal vs parenchymal<br>Pre-renal vs post-renal<br>Parenchymal vs post-renal	2.4 (1.8-3.5)	2.5 (1.8-4.3)	5.2 (2.3-8.4)	<0.001<br><br>0.208<br><0.001<br><0.001
Urea, *mg/dL*	130 (89-192)<br><br>Pre-renal vs parenchymal<br>Pre-renal vs post-renal<br>Parenchymal vs post-renal	140 (100-207.5)	93 (67.5-136)	140 (87-230)	<0.001<br><br><0.001<br>0.704<br><0.001
Previous CKD	263/565 (46.5)<br><br>Pre-renal vs parenchymal<br>Pre-renal vs post-renal<br>Parenchymal vs post-renal	154/310 (49.7)	41/121 (33.9)	68/134 (50.7)	0.006<br><br>0.011<br>0.918<br>0.016

Continuous variables are presented as median (IQR), and categorical variables as count (%) or proportion (%). Differences between groups were assessed using the Kruskal-Wallis test for continuous variables and Fisher's exact test for categorical variables. When overall significance was detected, pairwise comparisons were performed using the Dunn's multiple comparisons test.

Legend. AKI, acute kidney injury; CKD, chronic kidney disease.

Supplementary Table S3. Model comparison							
**Model**	**AUC**	**Youden-derived threshold**	**Sensitivity**	**Specificity**	**PPV**	**NPV**	**Accuracy**
Univariate (UCR alone)	0.794	37.7	79.7%	70.3%	90.9%	48.1%	77.7%
Age-adjusted	0.939	86%	82.6%	94.6%	98.3%	59.3%	85.1%
Multivariate	0.959	78.7%	89.1%	91.9%	97.6%	69.4%	89.7%

Legend. AUC, area under the curve; NPV, negative predictive value; PPV, positive predictive value; UCR, urea-to-creatinine ratio.
